# Rectal Surgery Made Easy

**Published:** 1887-11

**Authors:** 


### RECTAL SURGERY MADE EASY.

Edmund Andrews, M. D., LL. D., Professor of Medical Surgery in the Chicago Medical College, in an article with the above heading, in the Journal of the American Medical Association, February 5th, 1887, p. 148, says: A few days ago I learned and published the method of treating piles employed by certain itinerants. The secret was sold from one quack to another at a high price, and consists generally in the hypodermic injection of various mixtures of carbolic acid. The hopes of many physicians that the method might prove a useful one, were greatly dampened by the discovery of about eighteen deaths out of some 3.000 reported cases, and of very alarming symptoms in other instances; in short, it was the same experience as that which previously put a stop to the injection of venous enlargements in other parts of the body.

Of late the lake States are being treated to a new development; the little itinerant hypodermic syringe has budded and blossomed like Aaron's rod, and involved little boxes of instruments and little books full of secret instructions, in short, little "systems" of rectal surgery, whereby one of the authors says, "operations which would otherwise be difficult can be accomplished with but little skill.' The first style of boxes sells at a price varying from a hundred to a hundred and fifty dollars, according to the size of the purchaser's purse and credulity. They generally contain a hypodermic syringe and a rectal speculum, with a probe and a few other simple instruments, having some peculiarities of construction, but no special excellence. An equally good set of instruments for the purpose would cost about fourteen dollars at retail, in the ordinary instrument stores.

It is curious to notice the obvious efforts to combine a set of instruments in such a way that an ignorant purchaser may accomplish something with them, but shall not be able to do much harm. Generally there are no cutting instruments whatever, and the only sharp weapons are the hypoderic syringe and tanaculum. The chief formula for injection for hæmorrhoids is the following:

| | | |
|---|---|---|
| ℞. | Acid carbol.................................. | ℥ i |
| | Ol. olivæ...........................................fl. | ℥ v |
| | Zinci chloride.................................. | gr. i |

Armed with his little box and book the wayfaring doctor, though a fool, may read and practice, and perchance make money, if the crop of piles is good, although the science of the colleges never glance upon his calvarium.

Such an easy way to make money soon bred imitations. New boxes and new little books are in the market. Some contain several more instruments than those above mentioned, and yet are sold at half the price, being offered at about fifty dollars. There is, however, the same careful attention to the fact that the purchaser is expected to be such a fool that he cannot be trusted with edge tools, though some of the latter boxes contain the little blunt-pointed bistoury of Sims' uterine set, with an edge about half an inch long, for a purpose presently to be mentioned. One set which I examined contained the following instruments.

1. A small rectal speculum.
2. A hypodermic syringe.
3. Tenaculum.
4. Small Sims' uterine knife, blunt pointed and having an edge five-eighths of an inch long.
5. A Sims' flexible uterine probe for examing *fistulæ*.
6. A blunt hook to pull down the "pockets," presently to be described.
7. A similar blunt hook with two minute barbs to hold the pocket when it is so shallow as to slip from the smooth hook.
8. A scarifying probe, made so as to scratch the interior walls of a fistula to the depth of about a sixteenth of an inch. By a blundering combination of Greek and Latin in the same word, forbidden by the usages of educated men, it is called a "fistula-tome."
9. A flat scoop for clearing out the rectal pockets and pouches.
10. A ligature carrier, to facilitate ligating piles.

11. A three-ounce syringe.

14. A hard rubber tube for oiling the anus.

There are no scissors, probably because they could be dangerously used by ignorant purchasers.

The following were some of the printed directions in the box:

"*Radical Cure of Fistula in Ano.*—First trace fistula with flexible probe. Wash out the track with a 5-per cent. solution of 'hydrogen peroxide.' Then inject a 95-per cent. sol. of carbolic acid, plus equal quan. of 10 per cent. sol. of muriate of cocaine. Draw about 10 to 15 minims in the syringe. Push the flexible needle to the depth of the fistula, then inject slowly as you withdraw the needle. Within two hours inject oleum eucalyptus and glycerine, equal parts, and the operation is finished. Keep patient quiet for forty-eight hours."

*Hæmorrhoids.*—Hæmorrhoidal tumors should be injected with an eighth grain sol. of muriate of cocaine, plus an equal volume of 'phenol sodique,' use the injection from twenty minims to a drachm, according to size of tumor. It is seldom necessary to inject more than once or twice. This injection, deposited in two and three drops, making the punctures one inch apart over the rectum, will seldom fail to cure prolaps s of the rectum. Should be repeated two or three times.

During the treatment of hæmorrhoids or prolapsus, patient should take each night at bedtime a one-eighth grain pill of the solid extract leptandrin, and the parts should be kept annointed with 'ceratum taralinum,' once a day for a week after the operation. After operation on the rectum of a female, the uterus should be dilated, and after like operation on the male rectum, metalic should be passed as large as the uretha will admit; and in all cases where there are fissures of the anus, the sphincters should be forcibly dilated for ten seconds, while patient is under the influence of anæsthetic.

"With our improved instruments being expressly for treating the rectum, operations that would otherwise be difficult can be accomplished with little skill. Our speculum is the only one that is self-retaining, thereby enabling the operator to use both hands, at the same time exposing every part of the rectum, and causing the patient great pain by over-distention of the sphincters."

The "Phenol Sodique" is a French name for an article made and sold in Phildelphia. It is simply a solution of impure carbolic acid. The term "Ceratum Taralinum" is the result of an effort to construct a Latin name without knowing the language. The article to which it is applied is a blackish, untidy looking unguentum picis liquidæ, or tar ointment, but probably not made according to the official directions. Druggists inform me that it is sold as a proprietary article under the name "taroid."

A recent addition to these "systems of rectal surgery" consists in exploring the anus for the little pockets in the mucous membrane which exist normally just above the external sphincter, pulling them down with the blunt hook, and slitting them down with the Sims' knife.

Another edition consists in cutting off the little projections, or caruncles which are often found at the same part, under the preposterous claim that they are very injurious to the patient. The operators call these organs "pockets and fringes."

The possibilities of deception in this field are unlimited, since the patient can neither see the "pockets nor fringers" for himself, nor dispute their alleged pestiferous influence. This practice originated in a paper read before a Society of irregulars, entitled: "Rectal Pockets and Fringes." That the paper may be properly estimated I will call to memory here the structure of the lower rectum, as described by authors, both in anatomy and surgery: Morgagni described what any dissector can see, a recticulated columnar structure of the mucous membrane just above the external sphincter. These ridges or "columns of Morgagni," resemble the columnæ carneæ of the inner walls of the heart, but are very much smaller. Occasionally a probe will pass behind one of the columns, as under a bridge, but generally they are merely adherent pilasters, or ridges, running longitudinally, but somewhat branched. They are described not only by Morgagni, but "Curling on the Rectum," "Allen's Human Anatomy," "Kelsey on the Rectum," etc. Kelsey says, page 10: "Between the lower ends of the columnæ recti (columns of Morgagni) little arches are stretched, forming pouches. They are more developed in old people, and may retain small pieccs of fæces, and give rise to suppuration and abscesses."

Curling says, p. 6, that between the lower ends of these columnæ recti "the mucous membrane is slightly dilated, variously in different subjects, but in many to such an extent as to form small sacs, or pouches; and in the spaces between them there is a series of short projecting columnar processes about three-eighths of an inch in length."

The latter are analogous to the carunculæ myrtiformes of the vagina. The writer of the paper seized on these "pockets and fringes," as he called the pouches and caruncles, and declared that "our current literature contains little or no mention of them." In spite of the fact that they are natural organs, and therefore must have a use, he asserts that "they are more prolific of mischief than you would believe," and that "nowhere, so far as" he is "aware, are they well described or properly noticed," hence he advocates their destruction. The author of this paper bodly claiming old and well known anatomical facts, as almost a new discovery of his own, excited a good deal of controversy among the members of the Society, some supporting his claims and practice, and some roundly denouncing them.

The truth is that these structures are natural, and should be let alone in ordinary cases, but, like other organs, they occasionally become diseased, and require surgical interference. The itinerants, however, have added the "pockets and fringes" to their stock in trade, and believe that whenever they are found, the pockets must be split down and the caruncles, or "fringes," cut off. For this purpose the blunt hooks and the Sims' bistoury have been added to the wonderful little box, which enables them to "accomplish operations with but little skill."

The plan of treating fistulæ by injections of peroxide of hydrogen, followed by other antiseptics, is in principle the same as the iodine method formerly in vogue, which fell into disuse because of its uncertainty. There is no statistics to show whether this modification will do any better, but there can be no harm in trying it. It will probably be found, like the iodine plan, to fail in a large proportion of cases, but yet may sometimes be useful. It is obviously adopted for the itinerants, because their average ignorance is such that it would never do to trust them with the operation by incision. I shall be glad to receive letters from any physicians who have opportunity to know of the actual results.—*Technics*.

